# Supplementary material for: Consistent stoichiometric long-term relationships between nutrients and chlorophyll-a across shallow lakes
Source: Nat Commun. 2024 Jan 27;15:809. doi: 10.1038/s41467-024-45115-3 (PMC10821860; doi:10.1038/s41467-024-45115-3)
Supplement: Supplementary file 1 — Supplementary Information [file 41467_2024_45115_MOESM1_ESM.pdf]

Table of contents

1 Supplementary Notes **2**
1.1 Position of study lakes 2
1.2 Separate analyses of the correlation between nutrients and Chl-a for the Danish dataset and global data 2
1.3 Analysis of the correlation between nutrients and Chl-a for lake data without depth cut-off 5
1.4 Model intercepts and slopes 7
1.4.1 Slopes, 5-year simple moving averages and residuals 7
1.4.2 Intercepts, 5-year simple moving averages and residuals 10
1.5 Average dataset TN and TP concentrations versus average dataset TN:TP 14
1.6 SRP concentrations versus total phosphorus concentrations 15
1.7 Nitrate-N concentrations versus TN concentrations 16

2 Supplementary methods **16**
2.1 Detailed description of data-analysis steps 16
2.2 Approach to extract short-term and long-term signals using simple moving averages . 17
2.2.1 Choice of simple moving averages 17
2.2.2 Choice of lengths of simple moving averages 18
2.2.3 Approaches to test the usability of simple moving averages and the SAD approach to assess their ideal length 18
2.2.4 Why we analysed simple moving averages with a hierarchical bootstrap approach 18
2.3 Simulations of the simple-moving average approach 18
2.3.1 Simulation of a long-term signal 18
2.3.2 Simulation of a short-term signal combined with a long-term signal 22
2.4 Selecting the best simple-moving average length for the lake data 26
2.5 Selecting the best regression models 26
2.5.1 Choosing the type of regression model 26
2.5.2 Choice of model terms 27

Supplementary references **28**

1 Supplementary Notes

1.1 Position of study lakes

The shallow lakes (avg. depth < 6m) which contained sufficient data (five or more consecutive growing seasons with at least three observations per growing season) to calculate 5-year simple moving averages (SMA) were unevenly distributed globally (SFig. 1), with only one lake in New Zealand, and most lakes in the USA (SFig. 2), and in Denmark (SFig. 3).

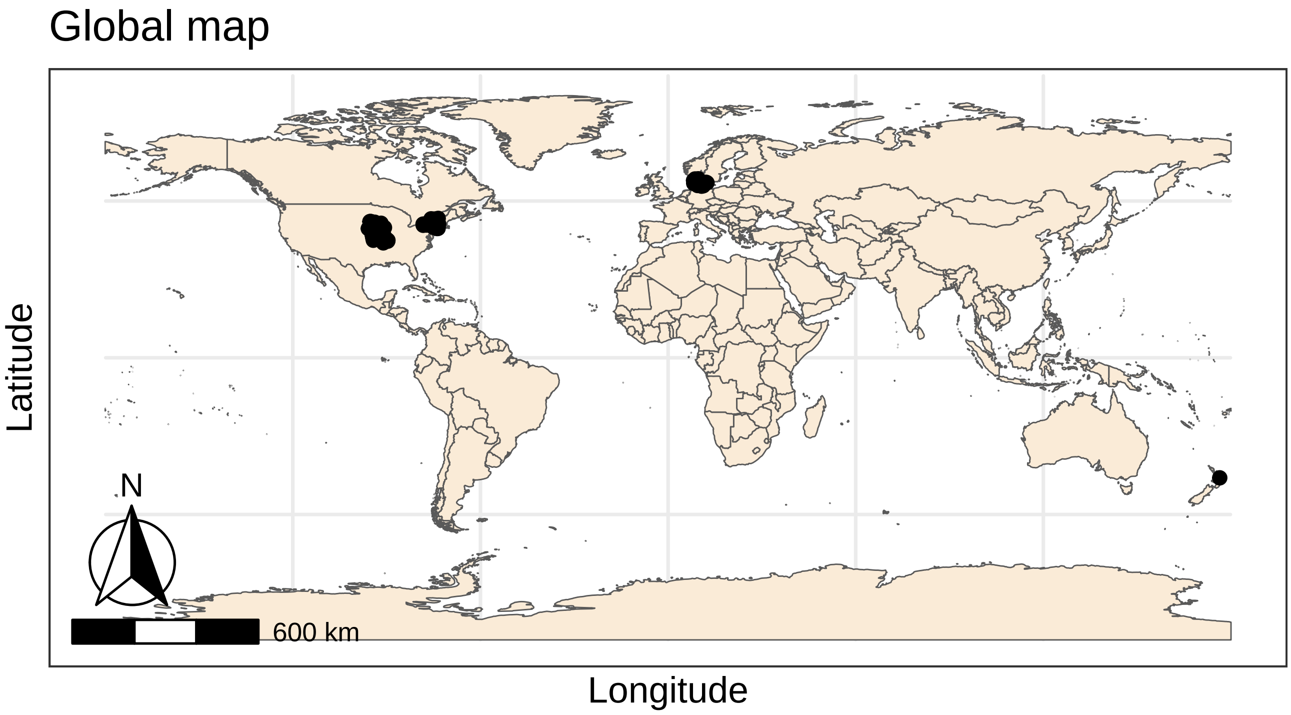

SFig. 1: Global map of lakes with 5-year simple moving average data. Lakes with sufficient data are situated in the US, Denmark and New Zealand. Maps have been generated by the authors in R with open-access map data from the rnaturalearth package.

1.2 Separate analyses of the correlation between nutrients and Chl-a for the Danish dataset and global data

To check whether the Danish dataset (Aarhus University, Danish Centre for Environment, https://odaforalle.au.dk) and global dataset[1] revealed the same response of Chl-a to TN or TP at the same TN:TP ratios, we conducted a separate analysis of the 5-year SMAs for both datasets using exactly the same statistical approach as for the entire dataset. As for the full dataset, the SMAs were randomly combined using the hierarchical bootstrap procedure (Section 2.1).

The separate analysis reveals two things. Due to the lower number of data within the separate analysis, the patterns are linked to somewhat higher uncertainty, hence higher variability of the model results on the y-axis (SFig. 4, SFig. 5). Still the two datasets give the exactly same answer as the full dataset presented in the main text for the pattern of R^2 (SFig. 4) and slope (SFig. 5) along the TN : TP axis.

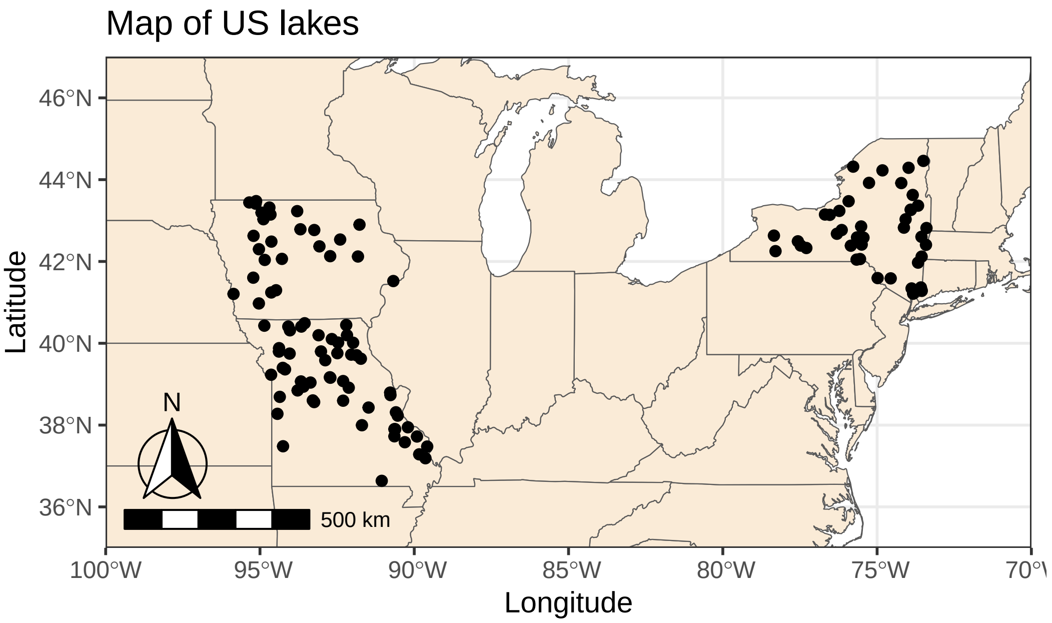

SFig. 2: Map of US lakes with 5-year simple moving average data. Maps have been generated by the authors in R with open-access map data from the rnaturalearth package.

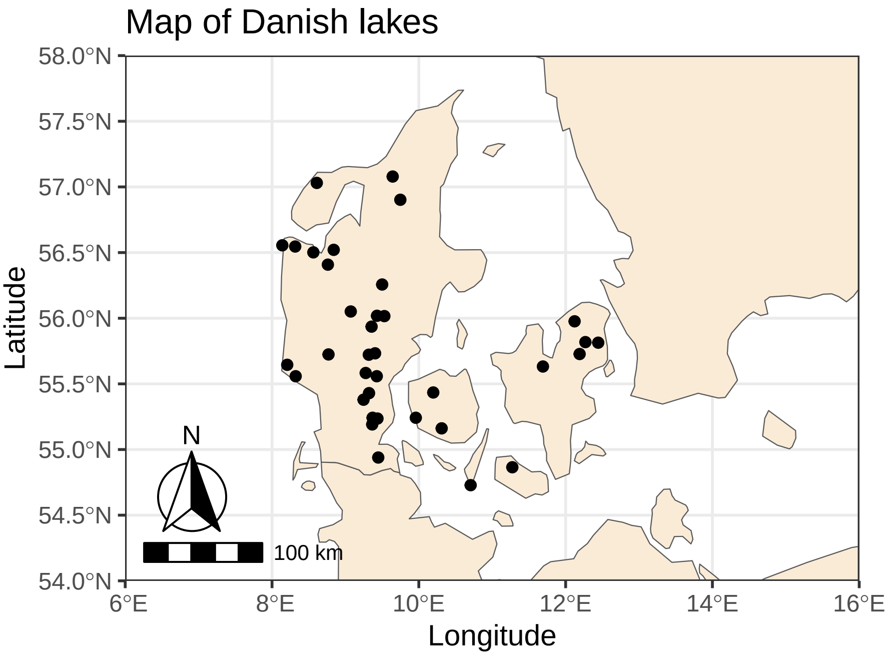

SFig. 3: Map of Danish lakes with 5-year simple moving average data. Maps have been generated by the authors in R with open-access map data from the rnaturalearth package.

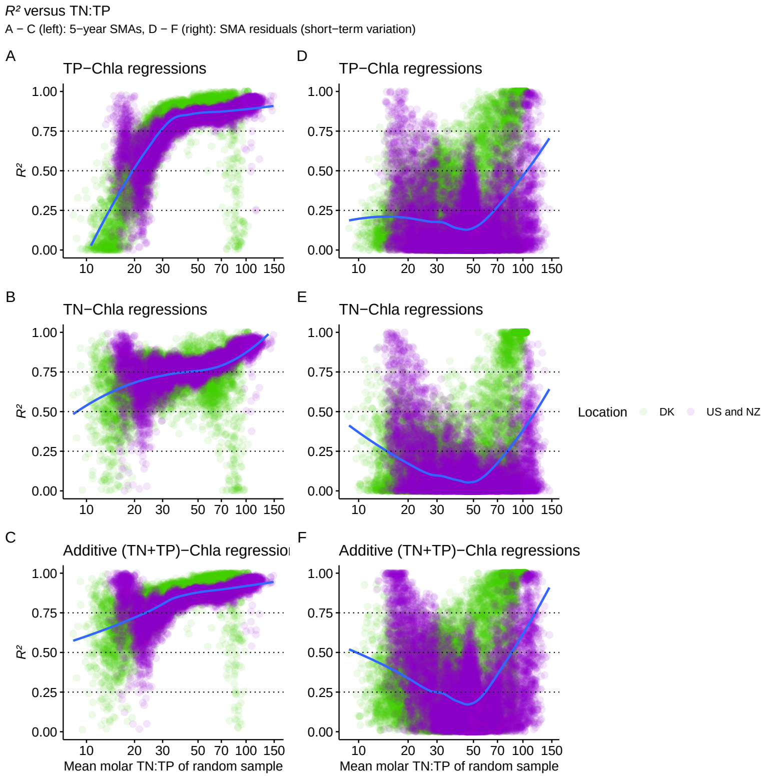

SFig. 4: Explained variance (R^2) of generalized linear models for the long-term varation based on 5-year simple moving average (SMA) (A - C), and the short-term variation based on the SMA residuals (D - F). Shown are results from generalised linear models with Gamma distributions for 5-year SMAs and linear models with Normal distributions for SMA residuals for total phosphorus (TP, mg / L, panels A, D), total nitrogen (TN, mg / L, panels B, E), or additive models of TP and TN (panels C, F) and chlorophyll a (Chla, µg / L). These are plotted against the mean molar TN : TP of each of randomly sampled dataset. The darker the points, the more overlapping solutions were found for R^2 by the bootstrap procedure (indicating the error of R^2). The orange line is the average response based on a LOESS function.

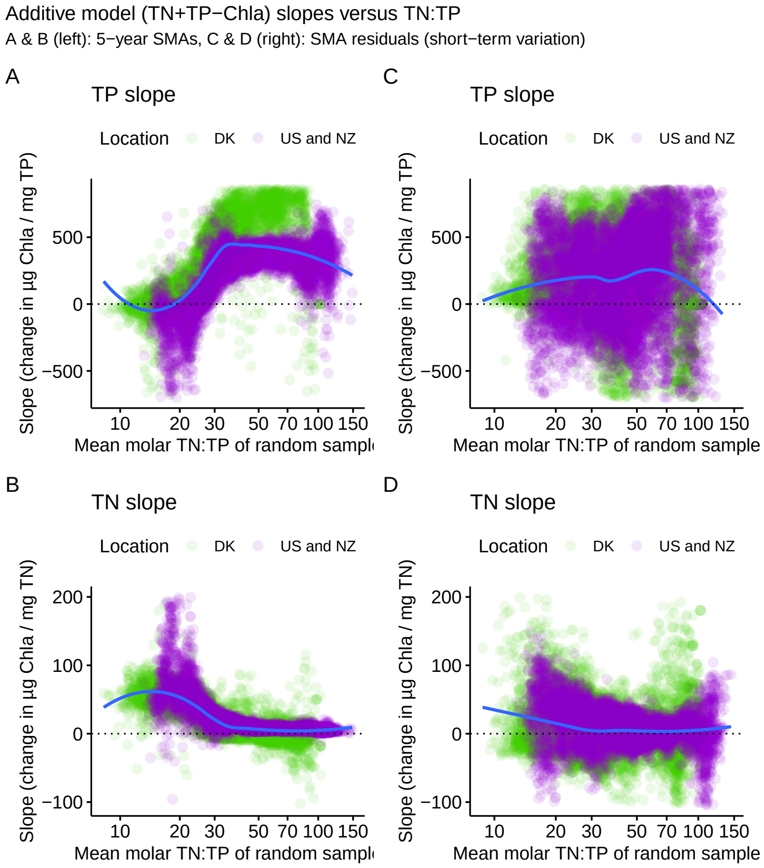

SFig. 5: Slopes of the additive models for long-term variation based on 5-year simple moving averages (SMA) (A & B) or short-term variation contained within SMA residuals (C & D) versus the mean molar TN : TP of each randomly sampled dataset. Shown are slopes from additive models between total phosphorus (TP, mg / L, panel A, C) or total nitrogen (TN, mg / L, panel B, D) and chlorophyll a (Chla, µg / L) versus the mean molar TN : TP of each of randomly sampled dataset. The darker the points, the more overlapping solutions were found for the slopes by the bootstrap procedure (indicating the error of the slopes). The orange line is the average response, based on a LOESS function. For easier comparison, we used the same y-axis range for the TP slopes of the 1-year SMA and SMA residuals, which removed 942 or extreme values for TP slopes of the the SMA residuals (see SI for full plot). Hence, N = 8252 iterations or n = 9142 for the TN slopes or the TP slopes of the 5-year SMAs, respectively (A & B); and n = 12858 or n = 13800 iterations for the TP and TN slopes of the SMA residuals, respectively (C & D).

1.3 Analysis of the correlation between nutrients and Chl-a for lake data without depth cut-off

When removing the depth cut-off of 6 m, we keep 1310 observations from 209 lakes with 5-yr SMA in the dataset. Thus, this includes 50 lakes more than for the data with depth cut-off. In other words, shallow lakes strongly dominate the dataset.

The TN:TP dependent R^2 patterns are less clear than for the data with depth cut-off and the uncertainty of R^2 (spread of model solutions) is high at the ends of the TN:TP gradient (SFig. 6). However, the R^2 of the models are also very high (SFig. 6). The slope results for the data without depth cut-off are highly similar to the models for the data with depth cut-off (SFig. 7).

The statistical results let us to conclude that the approach is highly promising for all lake types, irrespective of lake depth. Still, we keep the depth cut-off, since the dataset is dominated by shallow lakes. Thus, if different patterns would be the case for deeper lakes, we may not be able to see it and, from that deduct false conclusions on deeper lakes. Here, the low number of deeper lakes precludes us from a separate analysis of those. Furthermore, one would need to make sure that the water samples also do well represent the entire water column, something which we cannot be certain of. In contrast,

epilimnetic water samples do usually well represent the entire water column for shallow lakes < 6 m average depth[2].

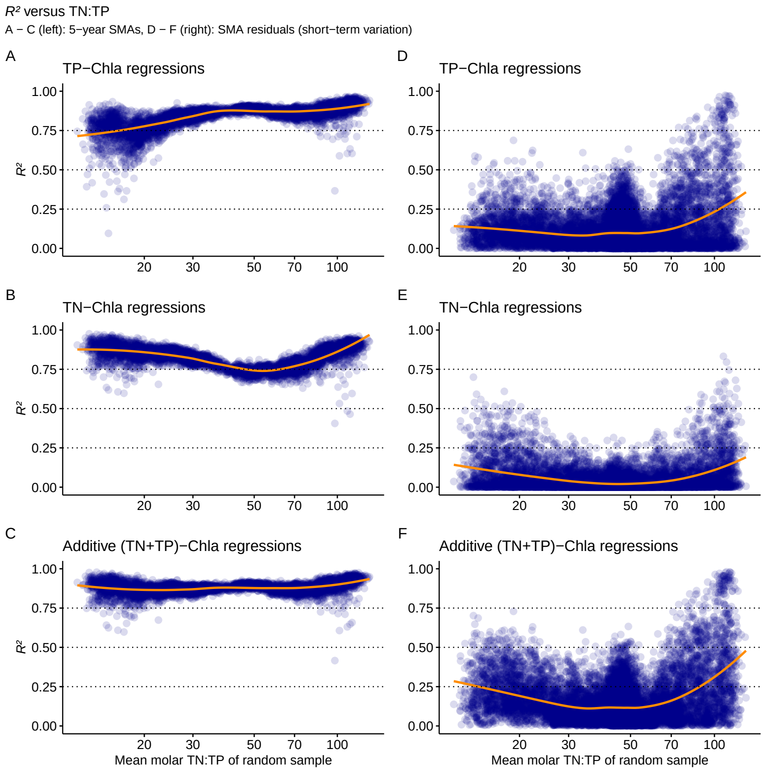

SFig. 6: Explained variance (R^2) of generalized linear models for the long-term varation based on 5-year simple moving average (SMA) (A - C), and the short-term variation (D - F) based on the SMA residuals. We included all data without using the depth cut-off. Shown are results from generalised linear models with Gamma distributions for 5-year SMAs and linear models with Normal distributions for SMA residuals for total phosphorus (TP, mg / L, panels A, D), total nitrogen (TN, mg / L, panels B, E), or additive models of TP and TN (panels C, F) and chlorophyll a (Chla, µg / L). These are plotted against the mean molar TN : TP of each of randomly sampled dataset. The darker the points, the more overlapping solutions were found for R^2 by the bootstrap procedure (indicating the error of R^2). The orange line is the average response based on a LOESS function.

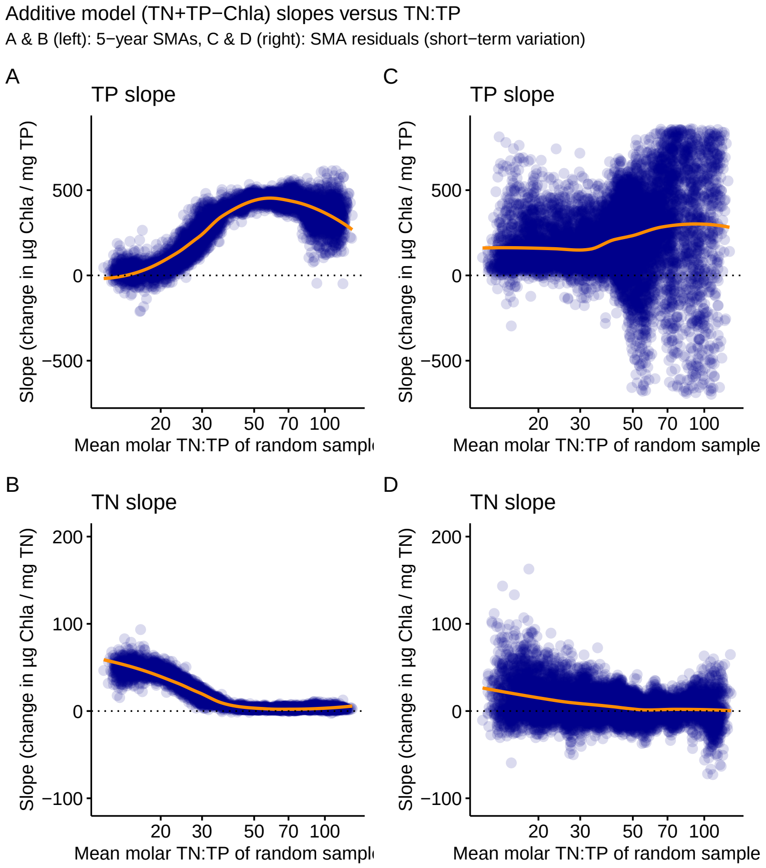

SFig. 7: Slopes of the additive models for long-term variation based on 5-year simple moving averages (SMA) (A & B) or short-term variation contained within SMA residuals (C & D) versus the mean molar TN : TP of each randomly sampled dataset. We included all data without using the depth cut-off. Shown are slopes from additive models between total phosphorus (TP, mg / L, panels A, C) or total nitrogen (TN, mg / L, panels B, D) and chlorophyll a (Chla, µg / L) versus the mean molar TN : TP of each of randomly sampled dataset. The darker the points, the more overlapping solutions were found for the slopes by the bootstrap procedure (indicating the error of the slopes). The orange line is the average response, based on a LOESS function.

1.4 Model intercepts and slopes

1.4.1 Slopes, 5-year simple moving averages and residuals

1.4.1.1 TN model slopes

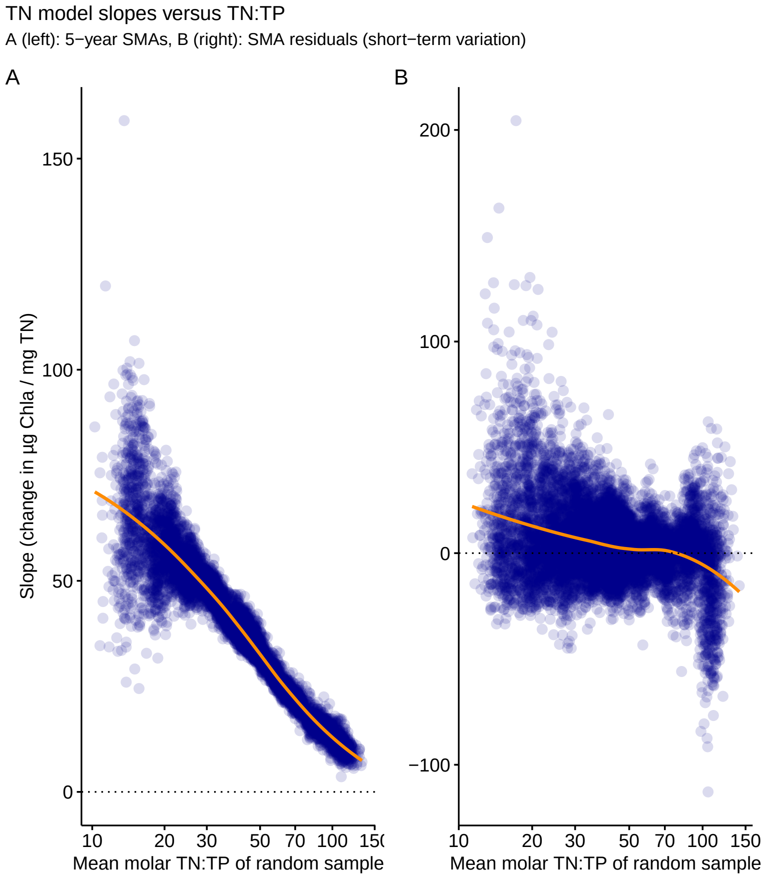

SFig. 8: Slopes of the TN models for long-term variation based on 5-year simple moving averages (SMA) (A) or short-term variation contained within SMA residuals (B) versus the mean molar TN : TP of each randomly sampled dataset. Shown are TN model between total nitrogen (TN, mg / L) and chlorophyll a (Chla, µg / L) versus the mean molar TN : TP of each of randomly sampled dataset. The darker the points, the more overlapping solutions were found for the slopes by the bootstrap procedure (indicating the error of the slopes). The orange line is the average response, based on a LOESS function.

1.4.1.2 TP model slopes

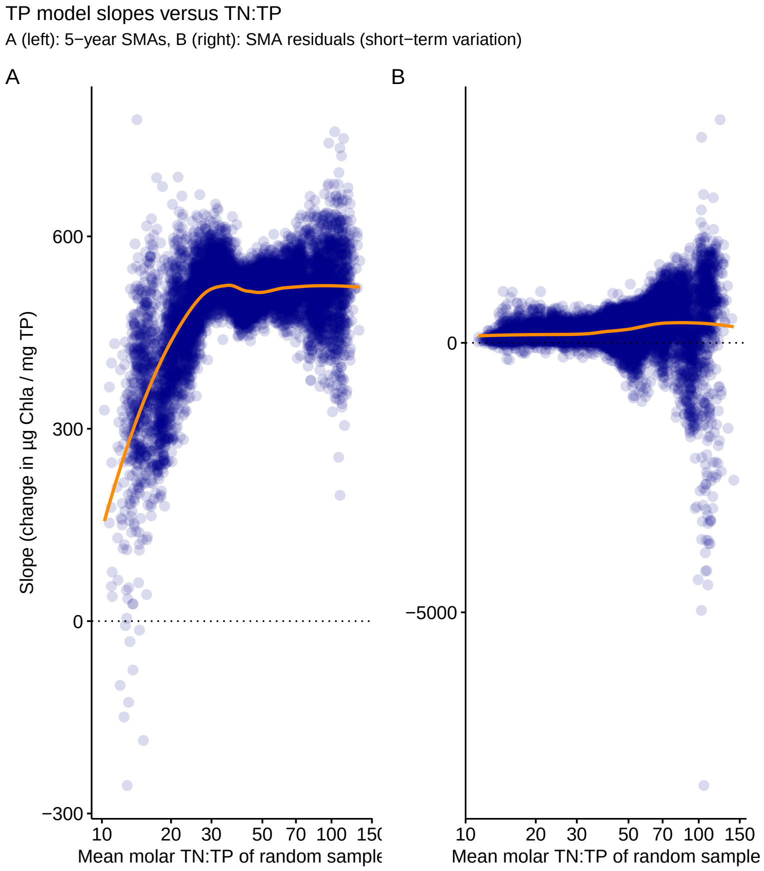

SFig. 9: Slopes of the TP models for long-term variation based on 5-year simple moving averages (SMA) (A) or short-term variation contained within SMA residuals (B) versus the mean molar TN : TP of each randomly sampled dataset. Shown are TP model slopes between total nitrogen (TP, mg / L) and chlorophyll a (Chla, µg / L) versus the mean molar TN : TP of each of randomly sampled dataset. The darker the points, the more overlapping solutions were found for the slopes by the bootstrap procedure (indicating the error of the slopes). The orange line is the average response, based on a LOESS function.

1.4.1.3 Additive model slopes

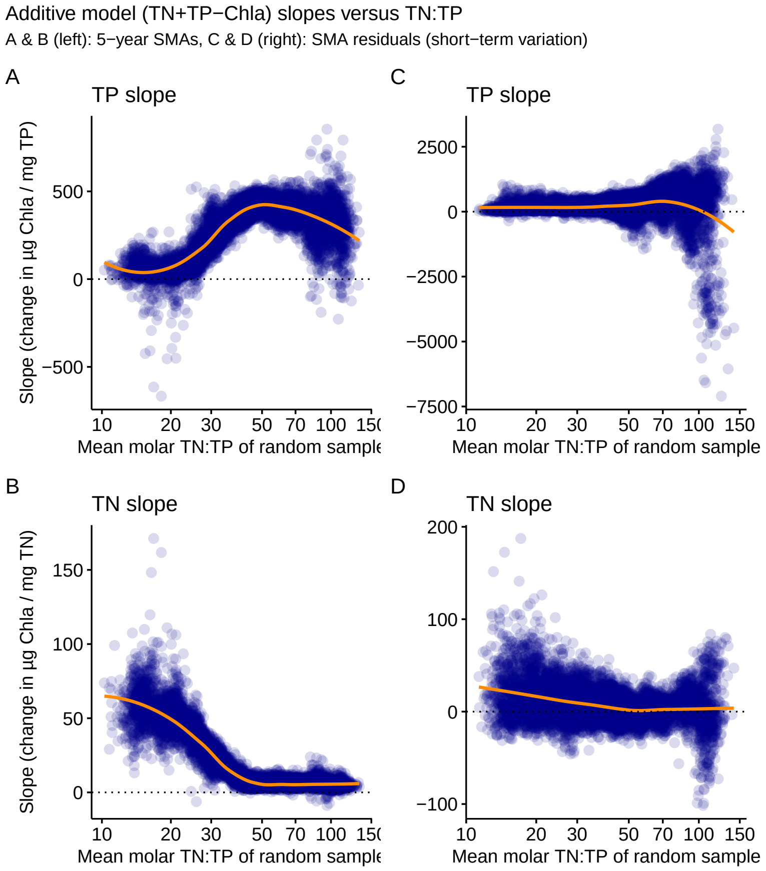

SFig. 10: Slopes of the additive models for long-term variation based on 5-year simple moving averages (SMA) (A & B) or short-term variation contained within SMA residuals (C & D) versus the mean molar TN : TP of each randomly sampled dataset. Shown are slopes from additive models between total phosphorus (TP, mg / L, panels A, C) or total nitrogen (TN, mg / L, panels B, D) and chlorophyll a (Chla, µg / L) versus the mean molar TN : TP of each of randomly sampled dataset. The darker the points, the more overlapping solutions were found for the slopes by the bootstrap procedure (indicating the error of the slopes). The orange line is the average response, based on a LOESS function.

1.4.2 Intercepts, 5-year simple moving averages and residuals

1.4.2.1 TN model intercepts

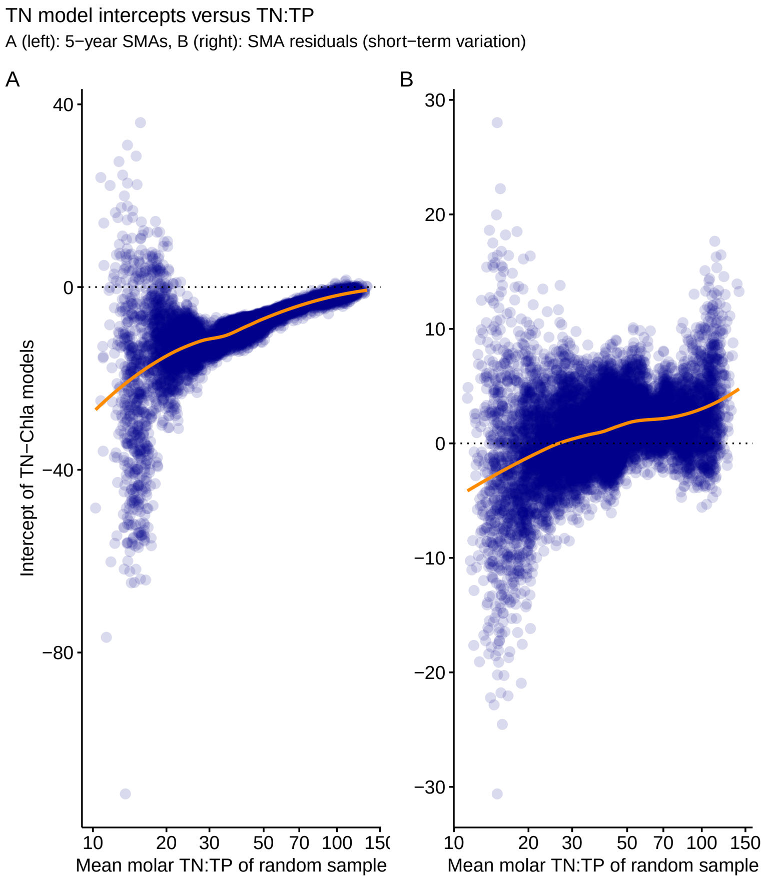

SFig. 11: Intercepts of the TN models for long-term variation based on 5-year simple moving averages (SMA) (A) or short-term variation contained within SMA residuals (B) versus the mean molar TN : TP of each randomly sampled dataset. Shown are TN model intercepts between total nitrogen (TN, mg / L) and chlorophyll a (Chla, µg / L) versus the mean molar TN : TP of each of randomly sampled dataset. The darker the points, the more overlapping solutions were found for the intercepts by the bootstrap procedure (indicating the error of the intercepts). The orange line is the average response, based on a LOESS function.

1.4.2.2 TP model intercepts

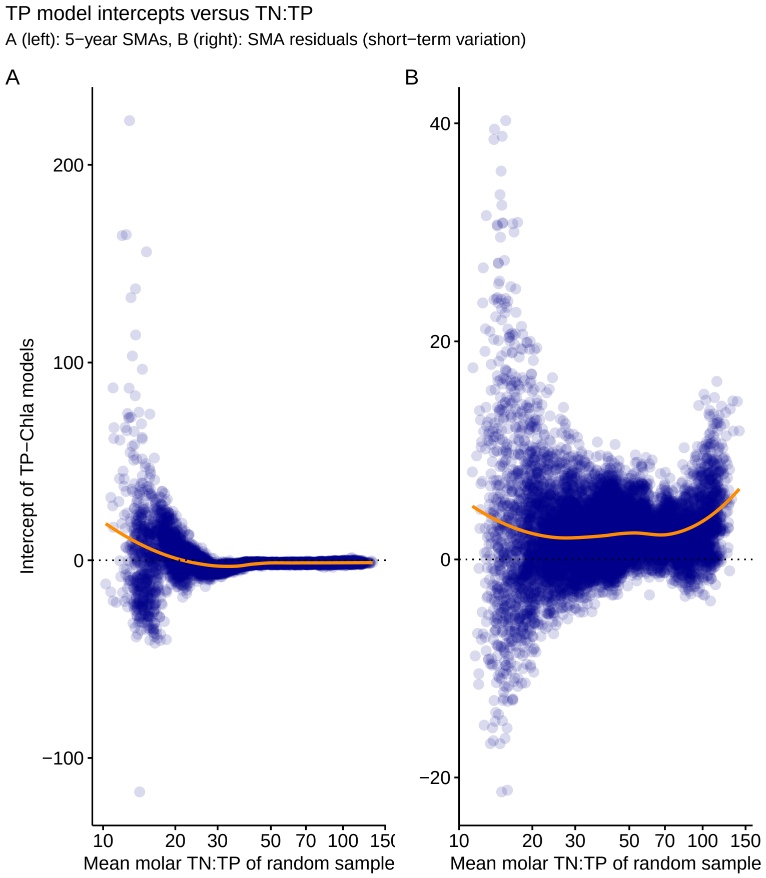

SFig. 12: Intercepts of the TP models for long-term variation based on 5-year simple moving averages (SMA) (A) or short-term variation contained within SMA residuals (B) versus the mean molar TN : TP of each randomly sampled dataset. Shown are TN model intercepts between total nitrogen (TP, mg / L) and chlorophyll a (Chla, µg / L) versus the mean molar TN : TP of each of randomly sampled dataset. The darker the points, the more overlapping solutions were found for the intercepts by the bootstrap procedure (indicating the error of the intercepts). The orange line is the average response, based on a LOESS function.

1.4.2.3 Additive model intercepts

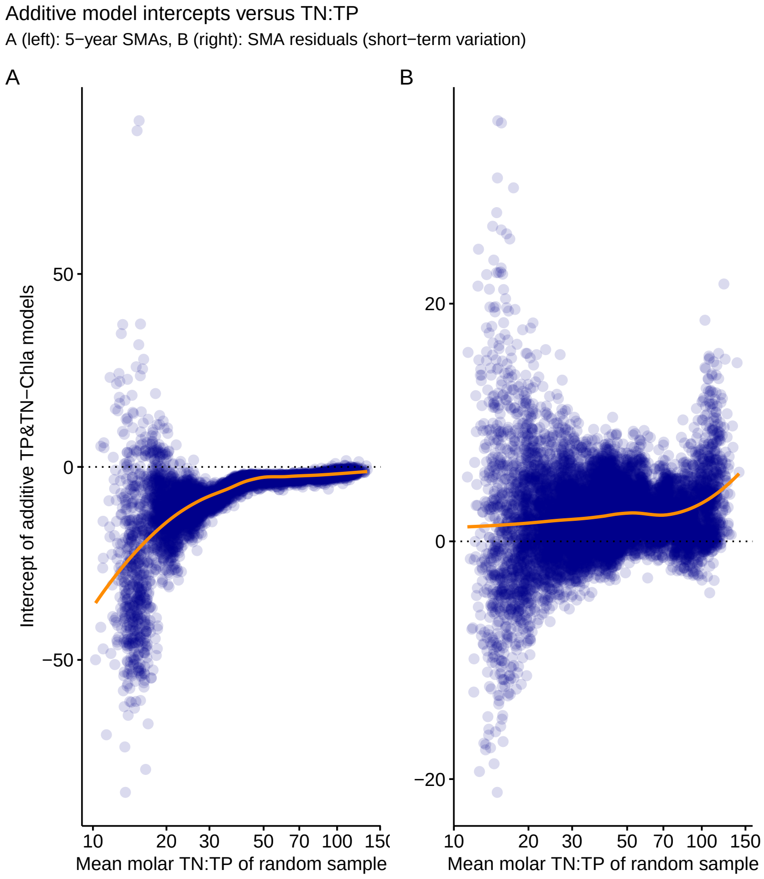

SFig. 13: Intercepts of the additive models for long-term variation based on 5-year simple moving averages (SMA) (A) or short-term variation contained within SMA residuals (B) versus the mean molar TN : TP of each randomly sampled dataset. Shown are intercepts from additive models between total phosphorus (TP, mg / L) or total nitrogen (TN, mg / L) and chlorophyll a (Chla, µg / L) versus the mean molar TN : TP of each of randomly sampled dataset. The darker the points, the more overlapping solutions were found for the intercepts by the bootstrap procedure (indicating the error of the intecepts). The orange line is the average response, based on a LOESS function.

1.5 Average dataset TN and TP concentrations versus average dataset TN:TP

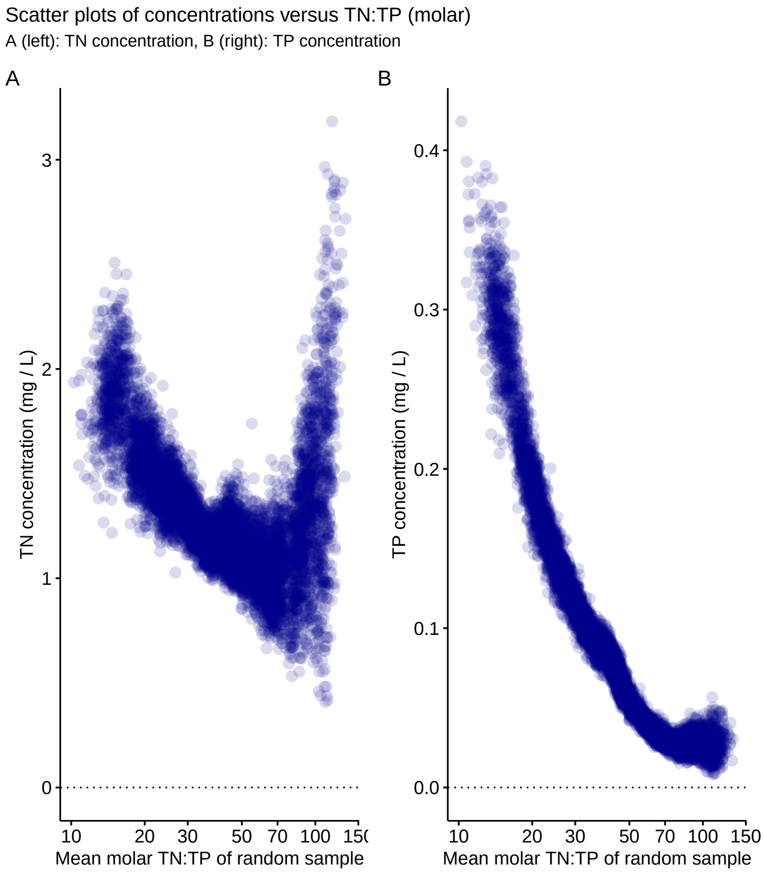

SFig. 14: Scatter plots of TN (panel A) and TP (panel B) concentrations verus TN:TP ratios for the 5-year SMA. The less transparent the points, the more overlapping data are displayed.

1.6 SRP concentrations versus total phosphorus concentrations

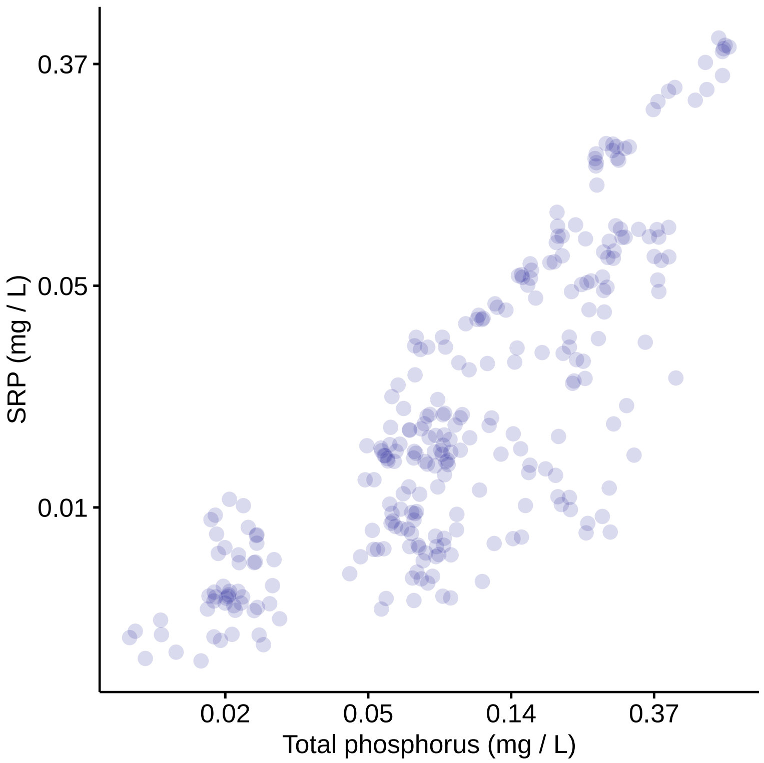

SFig. 15: Soluble reactive phosphorus (SRP) concentrations versus total phosphorus concentrations. SRP data were not available for every TN and TP observation, thus the plot contains less data than the plots of TN:TP, TN, TP and Chla. In this case 278 observations from 37 shallow lakes.

1.7 Nitrate-N concentrations versus TN concentrations

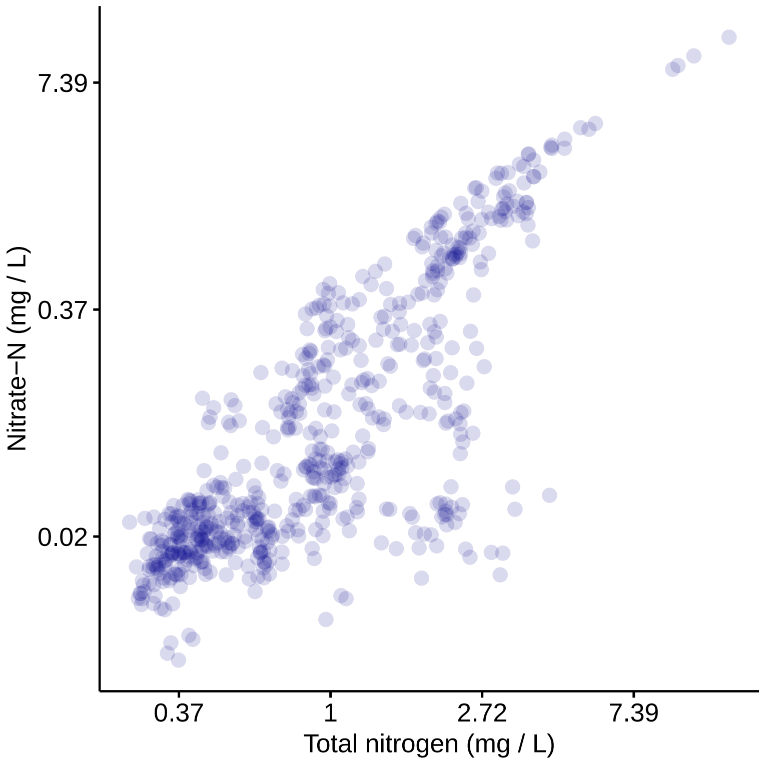

SFig. 16: Nitrate-N concentrations versus total phosphorus concentrations. Nitrate-N data were not available for every TN and TP observation, thus the plot contains less data than the plots of TN:TP, TN, TP and Chla. In this case 620 observations from 138 shallow lakes.

2 Supplementary methods

2.1 Detailed description of data-analysis steps

Here, we give a short rundown of the data-analysis steps (SFig. 17). In step 1, all possible moving averages are calculated for each lake, in step 2 all moving average data is combined. In step 3, the TN:TP ratio windows are defined with a minimum of ln molar TN:TP of 0 and a maximum of ln molar TN:TP of 7, a window width of ln molar TN:TP of 3 and a step of 0.1 ln molar TN:TP. The ratio windows are overlapping, hence, each data point can be drafted in multiple windows (step 3). These TN:TP ratio windows are applied to the data in step 4 (in this example a ln molar TN:TP between 2 and 5). All data filtered in step 4 is then bootstrapped at the lake level (randomly sampled with replacement, step 5a) and one observation for each randomly sampled lake is picked at random (step 5b).

This hierarchical bootsrap approach is the best way to reflect the structure of the original data. A simple, non-hierarchical bootstrap would favor lakes with more five-year means over lakes with less five-year means, simply because these make up a larger part of the data. Furthermore, sampling without replacement at lake level would result in five-year means from lakes with few data dominating the produced random dataset, as every lake would be sampled every time which then would result in high model leverage of five-year means from lakes with only few data. In contrast, the hierarchical procedure ensures that every lake has the same chance to end up in the randomly sampled bootstrap, in the second step it ensures that for each sampled lake, every five-year mean has the same chance to end up in the random dataset. These notions are in agreement with the findings of an assessment on how to properly resample hierarchical data by non-parametric bootstrap.[3]

For the generated random sample, three generalized linear models are calculated and kept, if the models converged (step 5c). This is done repeatedly (300 times, step 5d) for the data of each TN:TP ratio window to find all or most of the possible random combinations of lakes, with the aim to calculate

the error of the model estimates (pseudo R^2, AIC, intercept and slopes are used in the study).

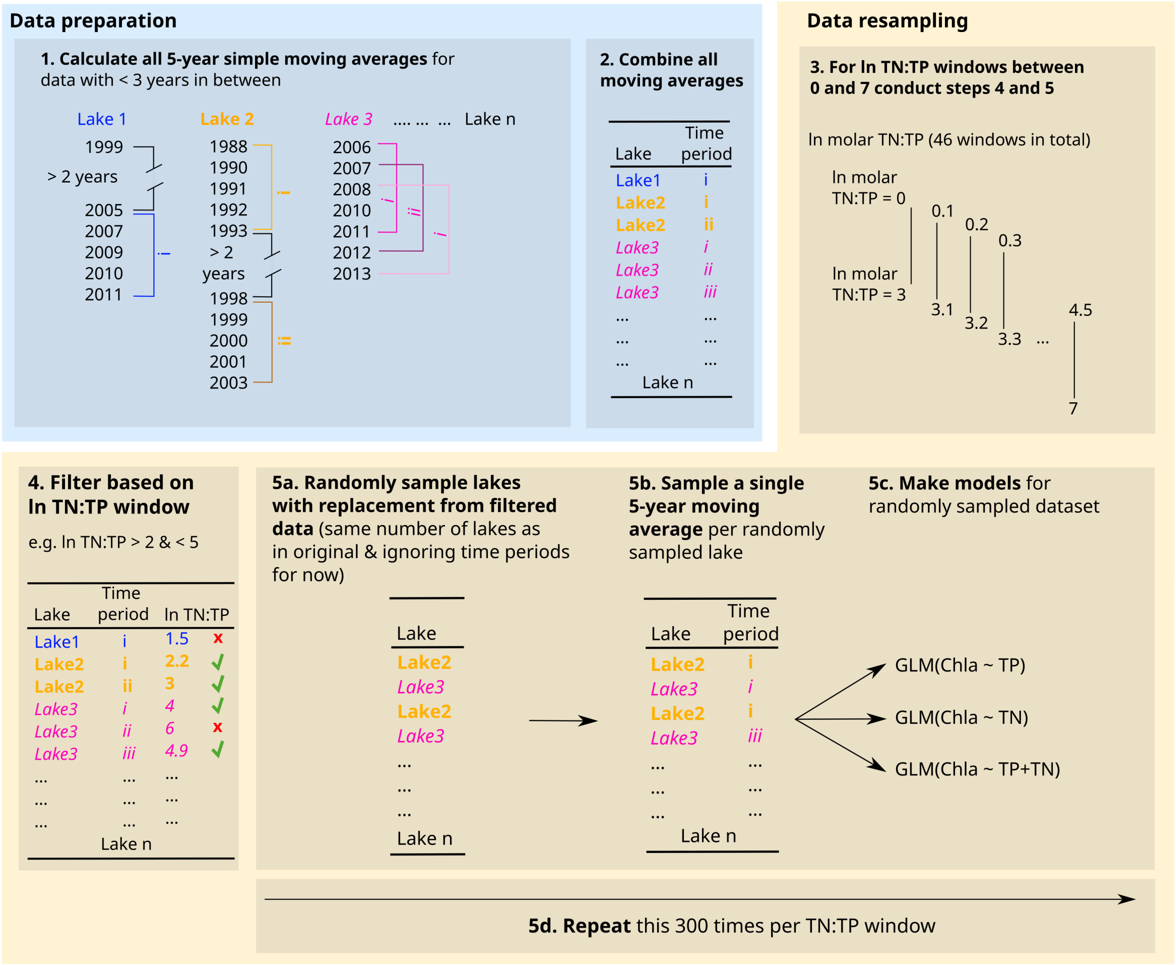

SFig. 17: A conceptual depiction of the data-analysis steps conducted within the study.

2.2 Approach to extract short-term and long-term signals using simple moving averages

2.2.1 Choice of simple moving averages

We chose to use SMAs to extract the short-term and long-term signals, because these are easy to compute and understand, because this approach compares well to more complex methods for long-term signal prediction.[4] Finally, we chose SMAs because short-term signals can easily be extracted by using their residuals, which is commonly done in e.g. economics, and has already once been done in limnology.[5]

2.2.1.1 How to extract of long-term and short-term signals from time series data using simple-moving averages

The SMA should contain the long-term signal and the residuals of the SMA should contain the short term signal. The SMA residuals are calculated as $observation - SMA$ for a given time point, e.g. an SMA for the years 2010 - 2015 would be positioned in 2013, and this SMA would be subtracted from the original observation of 2013.

Please note that only SMAs with odd numbers should be chosen if the SMA residual extraction should work. For even numbers, the time point of the SMA will not be an integer and the short-term observation cannot be aligned correctly (e.g., for a 4-year SMA from 2010 - 2014, the mean of the SMA would be the year 2012.5, which makes alignment of single-year observations impossible).

2.2.2 Choice of lengths of simple moving averages

Methods to extract different signals for different ranges of time series require to define the length of the signal to be extracted. SMAs are no exception to this rule. Here, an earlier study proposed to use a version of Akaike's information criterion, however, this approach gives one value for each time series, and the authors found the results of their approach highly variable depending on the individual time series[4]. Furthermore, this approach compares the predictive capability for different simple moving average lengths[4], instead we wanted to have the ideal length at which a long-term signal could be extracted if it existed.

To detect the ideal simple moving average length, we chose to use the sum of absoulte differences (SAD) between single-growing season values and SMAs. The SAD for a time series with a given simple moving average length is calculated as:

$$SAD = \sum_{i=1}^{k} |SMA_i - growing\ season\ observation_i| \tag{1}$$

Here, i is the year and k is the length of the time series. For each year i, the absolute difference between growing season observation (the mean of all values for a growing season) and value of the simple-moving average for the same year is calculated.

2.2.3 Approaches to test the usability of simple moving averages and the SAD approach to assess their ideal length

To test the capabilities of our SAD approach to calculate the best length for SMAs, and for SMAs to extract short-term and long-term signals from a time series in which systematic variation and random variation are mixed, we used two simulations and came to the following conclusions:

1. We could find the ideal simple moving average length and reconstruct a systematic long-term signal from a time series with random long-term random noise. Details on the simulation are found below (Section 2.3.1).
2. If a short-term signal of a correlation between nutrients and chlorophyll a would have been contained in addition to the long-term signal, we also likely would have found it, as we show with a second simulation. Details on the simulation are found below (Section 2.3.2).

Since the SAD approach worked well with simulated data, we applied it to real time series from our lakes, and show that 5-year SMAs are a good trade-off between simple moving average length and data availability (Section 2.4).

2.2.4 Why we analysed simple moving averages with a hierarchical bootstrap approach

SMAs have the drawback that they are a kind of auto-regressive model, where past data points and future data points affect the current value of the simple moving average[4], potentially affecting results of the regressions between nutrients and Chla. To account for this, we randomized data used for the correlations with the hierarchical bootstrap procedure described in Section 2.1 and shown conceptually in SFig. 17. Based on this approach, on average, only one observed simple moving average is picked from each lake, and, on average, each lake only appears only once in the dataset, making any effects of SMAs of the same time series interfering with each other impossible (SFig. 17). The same is true for the residuals of the SMAs.

2.3 Simulations of the simple-moving average approach

2.3.1 Simulation of a long-term signal

2.3.1.1 Generating the long-term signal

We mixed two signals, a long-term signal containing a perfect linear correlation between two variables to which we added a short-term signal, with different random noise for the two variables. Ideally we should be able to extract the linear correlation coefficients again from this mix, when using SMAs and the SAD approach.

First we created a long-term signal for a time series with 50 time points (resembling years), a variable x (could be e.g. a nutrient) and a variable y (could be phytoplankton biomass), which is dependent on x.

Here, variable x was created as:

$$x = (cos(pi * time_{1,2,...,50}/25) * 2) + 3 \tag{2}$$

where time is an integer from 1 to 50. With that, the long-term signal has a period length of 50, and a simple moving average with a length of 25 should be able to capture it again. The cosinus results were multiplied by 5 to which a constant of 3 was added to for better looking positive numbers.

Variable y was then calculated with a linear model:

$$y = 1 + x * 0.5 \tag{3}$$

Subsequently, we added random noise from a normal distribution to x and y, using the rnorm function with 50 samples in R with a mean = 0 and SD = 0.5 to create two independent random normal distributions, which we added to x and y. The results of the simulation establishment can be seen in SFig. 18. Without the random noise inserted by the normal distributions, $r^2 = 1$, slope = 0.5, and intercept = 1 for the correlation between x and y. By adding the random noise, the r^2 between x and y is lowered to 0.52, and the intercept and slope are changed randomly (intercept = 1.46, slope = 0.37). The scatter plot reveals clear variability around the linear model line (SFig. 18c).

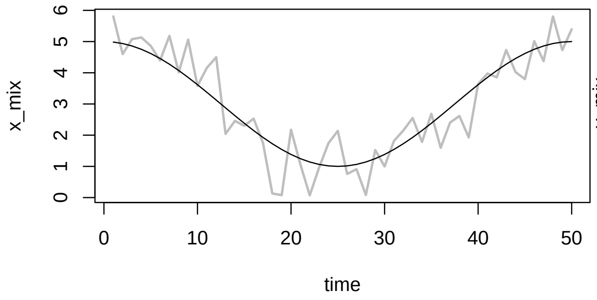

(a) x time series

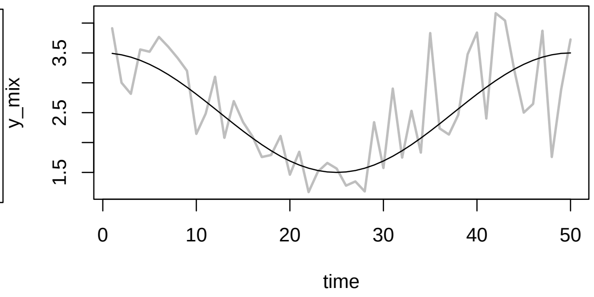

(b) y time series

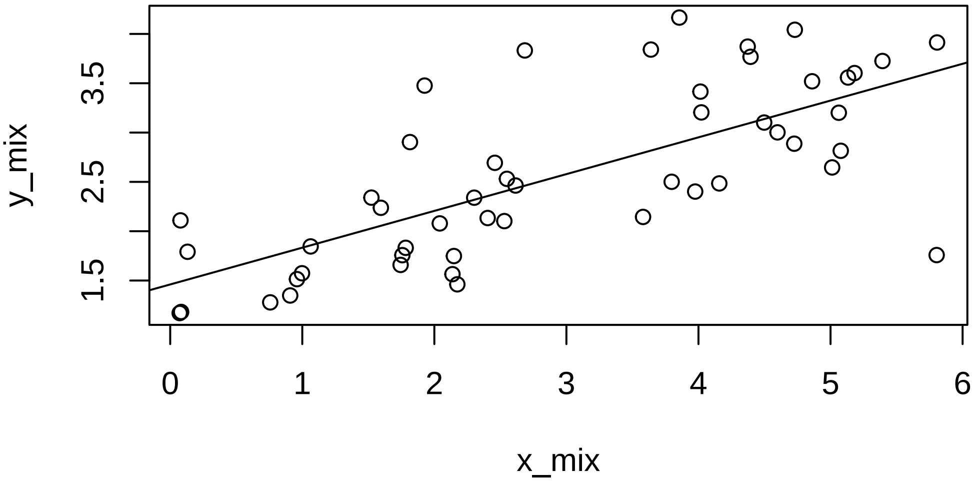

(c) Linear regression between randomized x and y

SFig. 18: Long-term signal (black line) and long-term plus randomized short-term signal for variable x and y (grey line), and linear regression between randomized variables x and y.

2.3.1.2 Estimating the ideal simple moving average length

We calculated the SAD for simple-moving average lengths of k = 2 - 40 (k equals the number of time steps) for the simulated x and y. In our simulations, we always see the unimodal pattern appearing

for the SAD (SFig. 19a, SFig. 19b), where the SMA at the very lower end of the SAD incline capture still considerable short-term variation, and the SMA shortly before, at or after the SAD peak remove part of the long term signal (SFig. 19c, SFig. 19d). We, recommend using the SMA in the middle of the SAD incline, clearly before the SAD peak. In this case we chose the SMA with k = 15 (here one could also use 13 or 17), which largely follows the long-term signal without capturing too much of the random short term signal (SFig. 19c, SFig. 19d).

2.3.1.3 Reconstructing the long-term signal in the relationship between x and y

We chose SMA with k = 15 (SFig. 19c, SFig. 19d) and found that the correlation of the SMA of x and y improved ($r^2 = 0.9$) (SFig. 20) relative to the correlation of the x and y with random noise ($r^2 = 0.52$) also reported in Section 2.3.1.1 (and shown in SFig. 18c). Be aware, this k is ideal for the simulated data and is different from the ideal SMA length used for the observed lake data in main manuscript (which has k = 5). Below, we describe in detail, how we arrive at a k = 5 for the lake data (Section 2.4).

Then, we tested whether we could reconstruct with the SMA data the original slope and intercept of the linear model for x and y defined in Equation 3. For the linear regression SMA of x and y with k = 15, we found a slope = 0.49 and an intercept = 1.19. This was close to the slope = 0.5 and intercept = 1 of the original linear model (Equation 3), and much closer than the slope = 0.37 and intercept = 1.46 of the regression of x and y with random noise (SFig. 18c).

We conclude that can find an approbriate SMA length. With that we can successfully reconstruct long-term signals in the correlation of two variables, such as TN and Chla or TP and Chla.

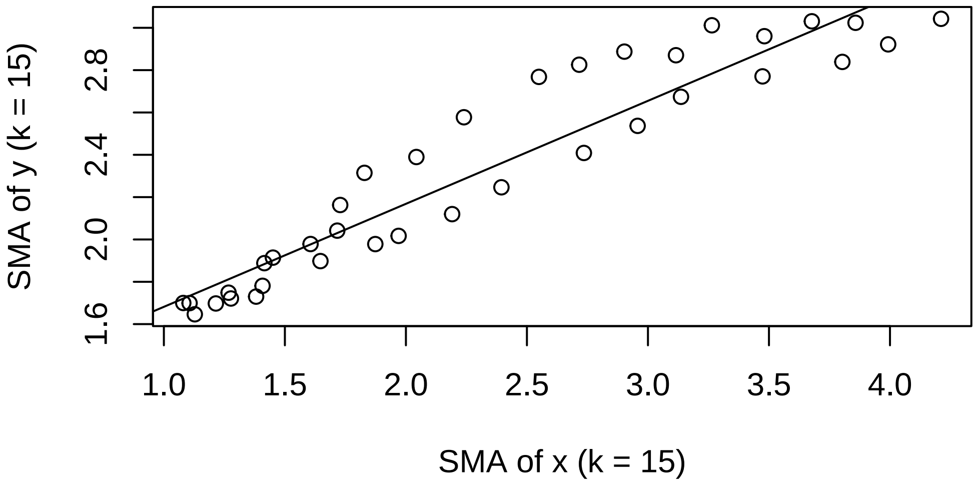

SFig. 20: Linear regression between SMAs with length (k) = 15 of x and y

2.3.1.4 Assessing whether we falsely detect a systematic short-term signal

Since we only included a random short-term signal, the residuals of SMA with k = 15 should only provide random noise for the correlation between x and y. Here, the residuals were calculated as simulated x and y (with random noise) minus the SMA of x and y (see for details)

For linear regression of the SMA residuals of x and y, the $r^2 = 0.1141812$, and the scatter plot also indicates no signal of a regression (SFig. 21)

This analysis shows that our approach does not falsely detect a short-term signal.

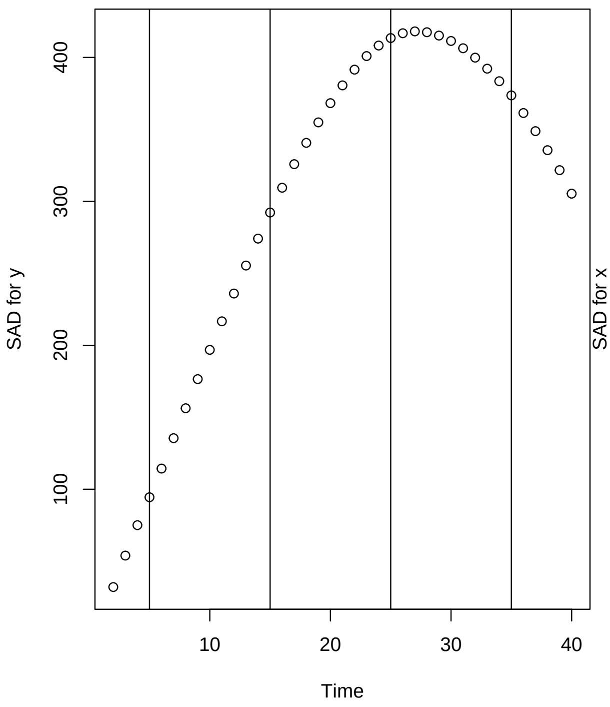

(a) SAD for different SMAs of y (vertical lines)

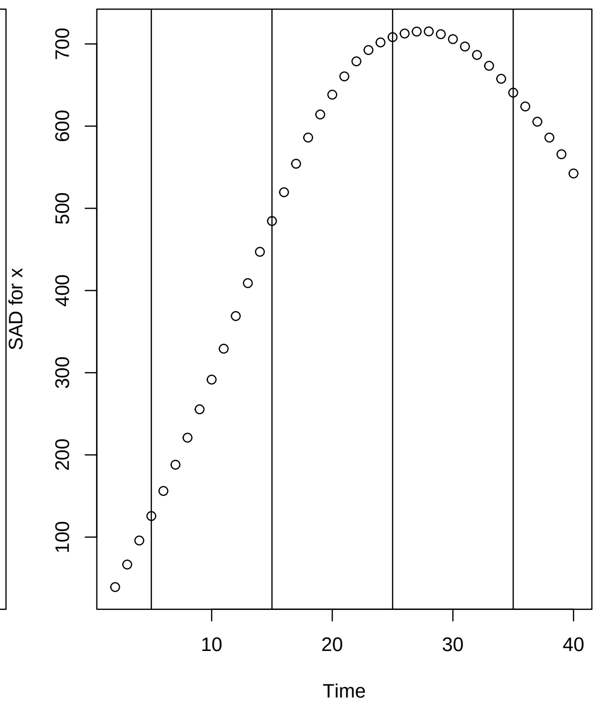

(b) SAD for different SMAs of x (vertical lines)

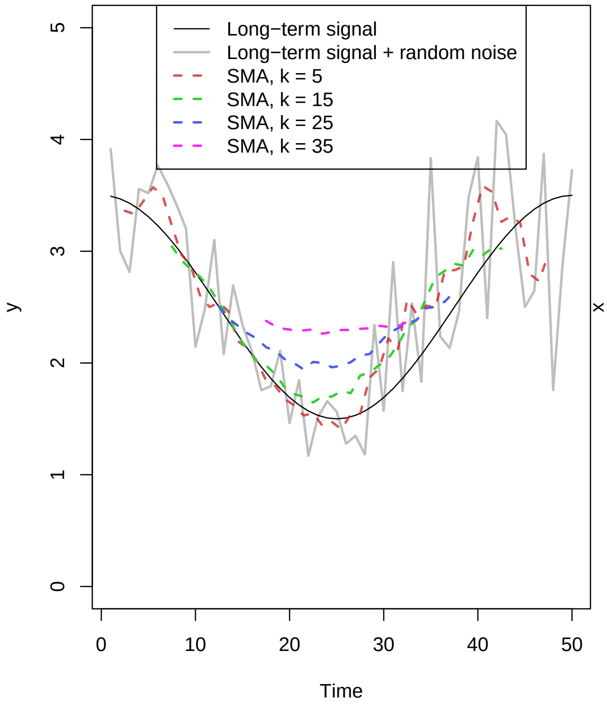

(c) y time series with SMA of different length k

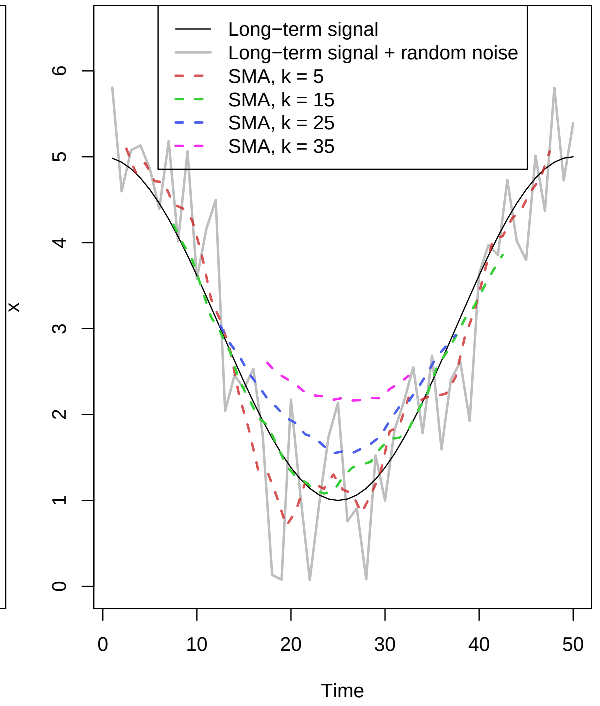

(d) x time series with SMA of different length k

SFig. 19: Sums of absolute differences (SAD) for y and x, as well as plots containing the long-term signal, the randomized long-term signal, and simple moving averages (SMAs) of different lengths (k).

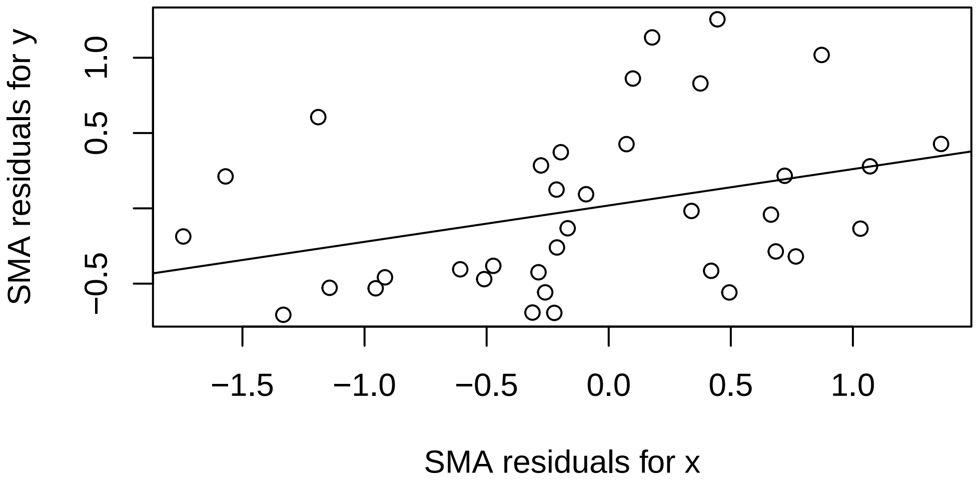

SFig. 21: Scatter plot of x and y residuals, calculated from the simulated x and y minus the SMA (k = 15) of x and y

2.3.2 Simulation of a short-term signal combined with a long-term signal

Above we show that we can successfully reconstruct a long-term signal and do not falsely detect a short-term signal (Section 2.3.1).

Here, we assess whether we would also detect a short-term signal if it existed in the data using the SMA calculation and residual calculation already described above. We do not repeat the description of the general approach here, please see our approach description above for further details (Section 2.2). Furthermore, in this simulation, we repeat the long-term simulation, which is described in detail above (Section 2.3.1).

2.3.2.1 Generating the signals

We again used the long term relationship between x and y as described in Section 2.3.1.1. Specifically, we calculated the long-term signal (x_{long} and y_{long}) based on Equation 2 and Equation 3 (SFig. 22a). To this, we added a short-term signal for x (x_{short}) and y (y_{short}) based on a regression with a negative slope. To achieve this, we first created a single normal distribution $N_{short} = N(0, 0.5)$ with 50 values (one for each time step). Then we calculated $x_{short} = -1 * N_{short}$ and $y_{short} = 2 * N_{short}$, giving an intercept = 0 and a slope = -2 for the regression between x_{short} and y_{short} (SFig. 22b).

We combined the short-term and long-term signal as $x_{mix} < -x_{short} + x_{long}$ (SFig. 23a) and $y_{mix} < -y_{short} + y_{long}$ (SFig. 23b). The mixed signals x_{mix} and y_{mix} exhibited only a weak relationship (SFig. 23c).

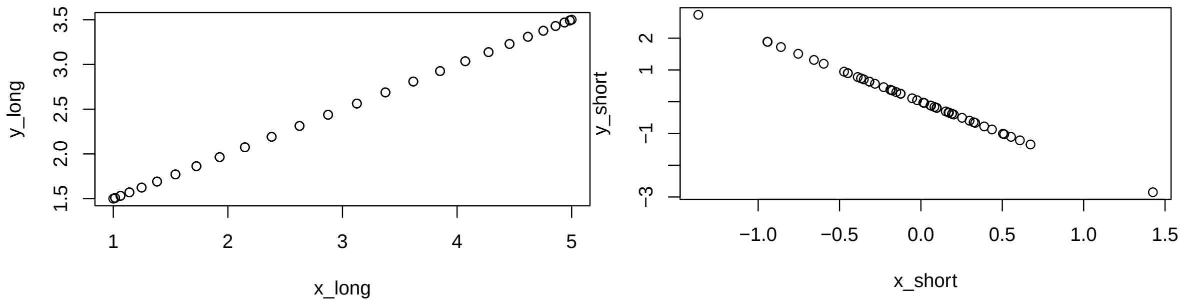

(a) Long-term signal

(b) Short-term signal

SFig. 22: Scatter plot of x and y for the long-term and short-term signal

2.3.2.2 Estimating the ideal simple moving average length

The ideal SMA length is again approximately k = 15, as with longer SMAs, the long-term variation gets dampened, and shorter SMAs (here k = 5) remove less short-term variation (SFig. 24c & SFig. 24d).

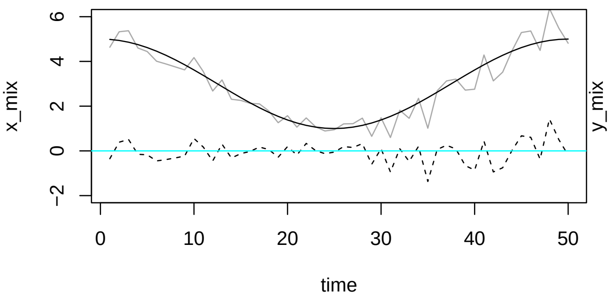

(a) x time series

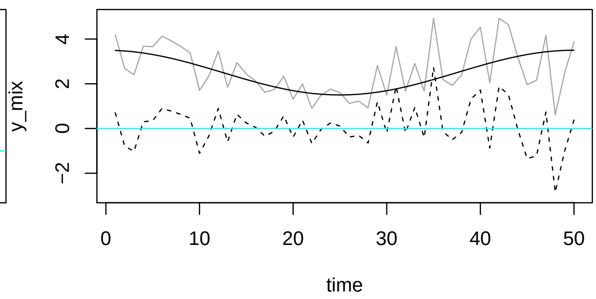

(b) y time series

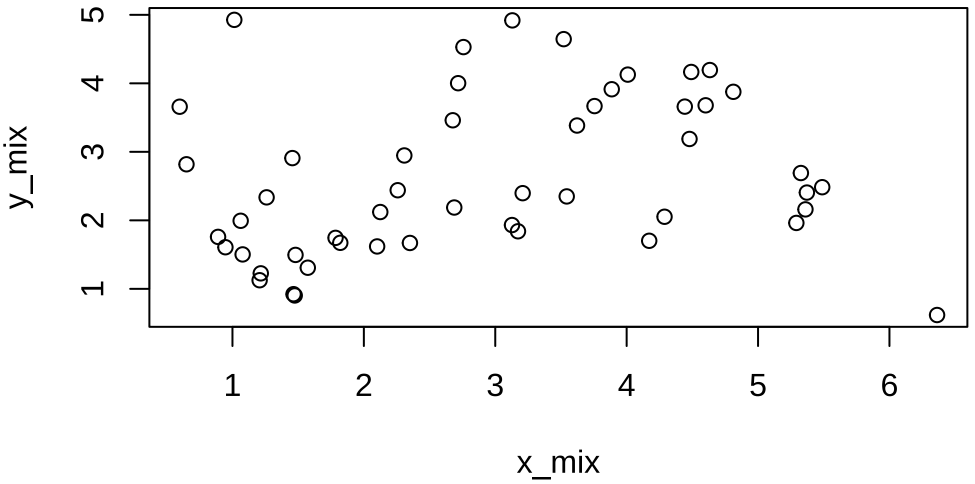

(c) Linear regression between mixed signals of x and y

SFig. 23: Long-term signal (black line), short-term signal (black, dashed line) and mixed signal (grey line) for variable over time, and linear regression between mixed signals of x and y.

On the SAD curve a SMA with k = 15 is on the middle of the incline before the SAD peak (SFig. 24a & SFig. 24b)

2.3.2.3 Reconstructing the long-term and short-term signal in the relationship between x and y

Neither the short nor the long signal was visible in the mixed signal ($r^2 = 0.06$) (SFig. 23c). However, the long-term signal could be reconstructed to a large extent using SMAs. Here, the SMA with k = 15 gave a $r^2 = 0.76$ for the regression between x and y (SFig. 25), and we estimated an intercept = 1.22 and a slope = 0.51, which is close to the actual intercept of 1 and slope = 0.5 of the regression between x_{long} and y_{long}.

We could also reconstruct the short term signal with a $r^2 = 0.83$ (SFig. 26). Here, regressing the SMA residuals yielded an intercept = -0.22 and slope = -1.88 between x and y, which is reasonably close to the true slope = -2 and intercept = 0 of x_{short} and y_{short}.

We conclude that our approach can reconstruct mixed short-term and long-term signals in the regression of two variables. This strongly suggests that our approach would have found a short-term signal of the relationship between nutrients and Chla, if it existed.

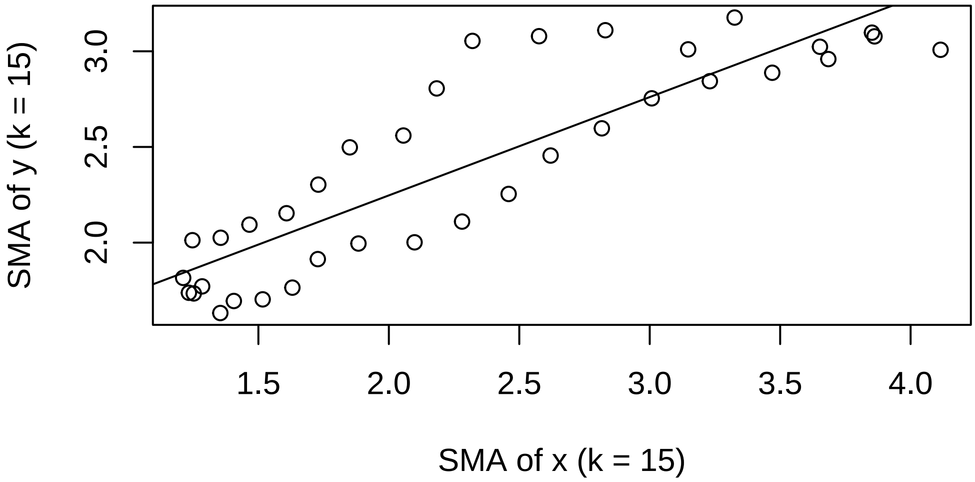

SFig. 25: Linear regression between SMAs with length (k) = 15 of x and y

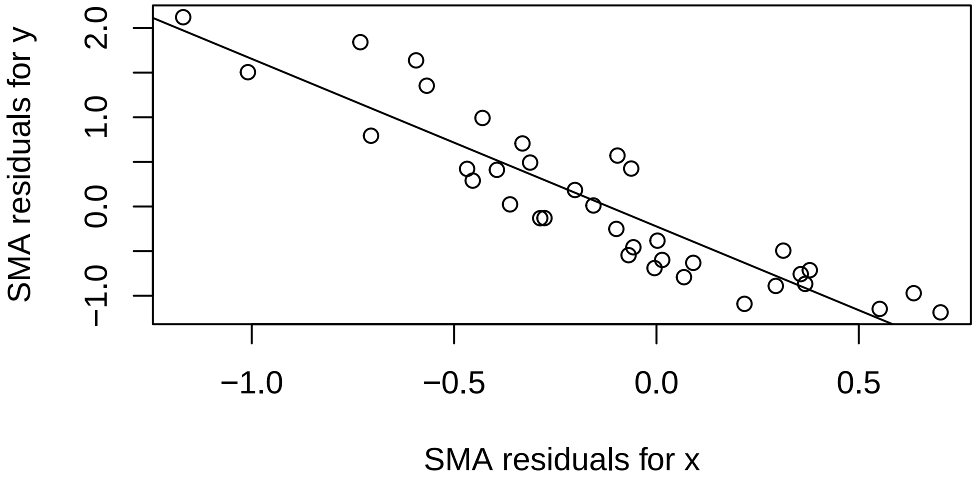

SFig. 26: Scatter plot of x and y SMA residuals, calculated from the simulated x and y minus the SMA (k = 15) of x and y

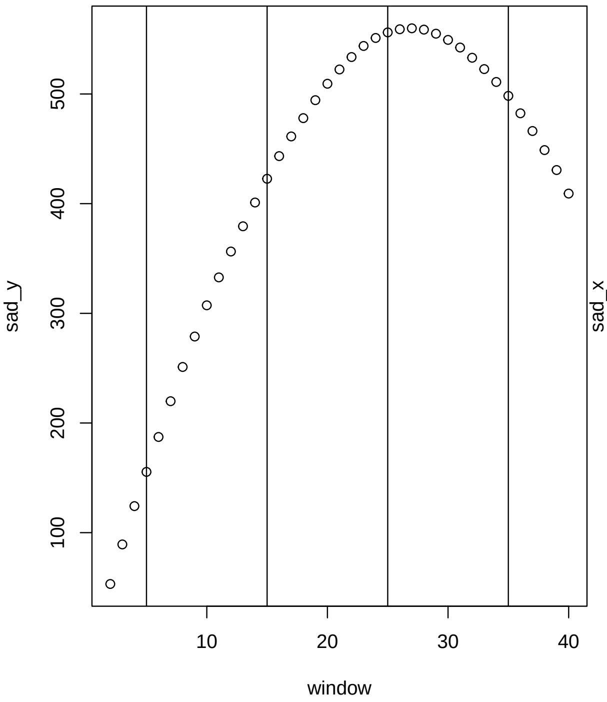

(a) SAD for different SMAs of y (vertical lines)

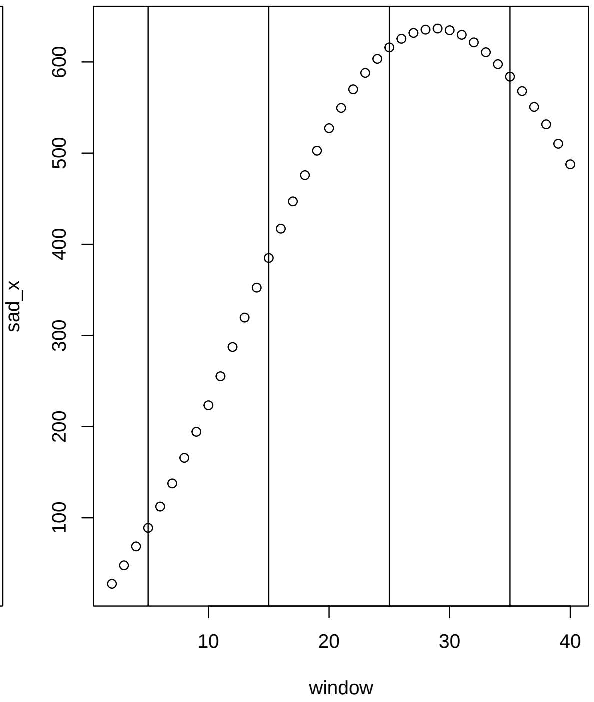

(b) SAD for different SMAs of x (vertical lines)

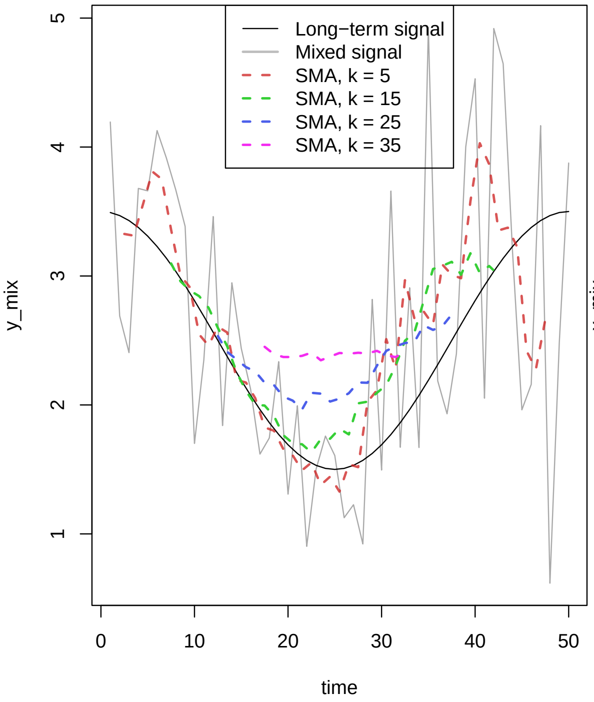

(c) y time series with SMA of different length k

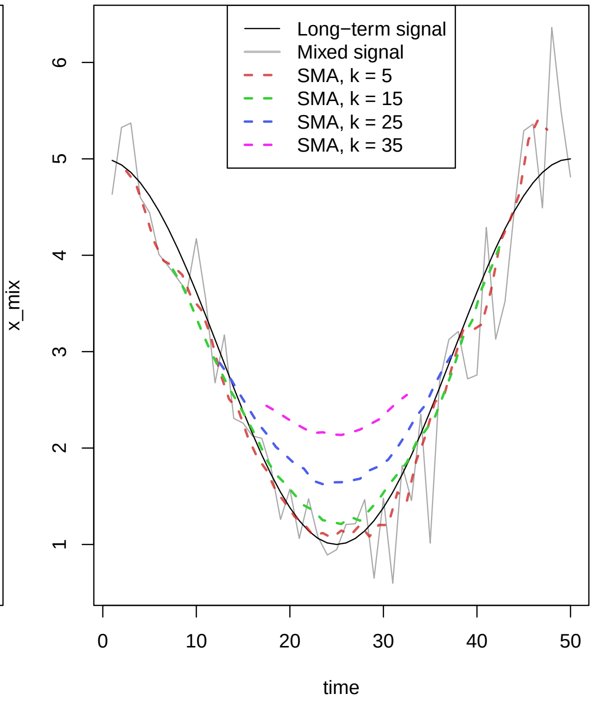

(d) x time series with SMA of different length k

SFig. 24: Sums of absolute differences (SAD) for y and x, as well as plots containing the long-term signal, the randomized long-term signal, and simple moving averages (SMAs) of different lengths (k).

2.4 Selecting the best simple-moving average length for the lake data

We use the SAD approach described above (Section 2.2.2) to select the ideal SMA length for the real lake data. As can be seen in the simulations with a only long-term, or a mixed long-term and short-term signal, the ideal SMA length – i.e. the SMA length where the short-term variation is largely removed, but the long-term variation is not dampened – is at the range of SAD before the peak but not at the lower end of the incline (see SMA plots of simulation results in Section 2.3.1.2 and Section 2.3.2.2).

The SAD of the real lake data shows exactly the same pattern as the simulated data with an increase, peak and subsequent decrease with longer SMAs. Here the average SAD peak was at an SMA length of 7 to 11 years, but some time series already showed a decline in SAD with SMA lengths of more than 7 years (SFig. 27). We therefore considered a 5-year SMA to be ideal, as this was still on the ascent of the SAD for all time series. A 5-year SMA also allowed us to retain a relatively high number of shallow lakes (SFig. 27).

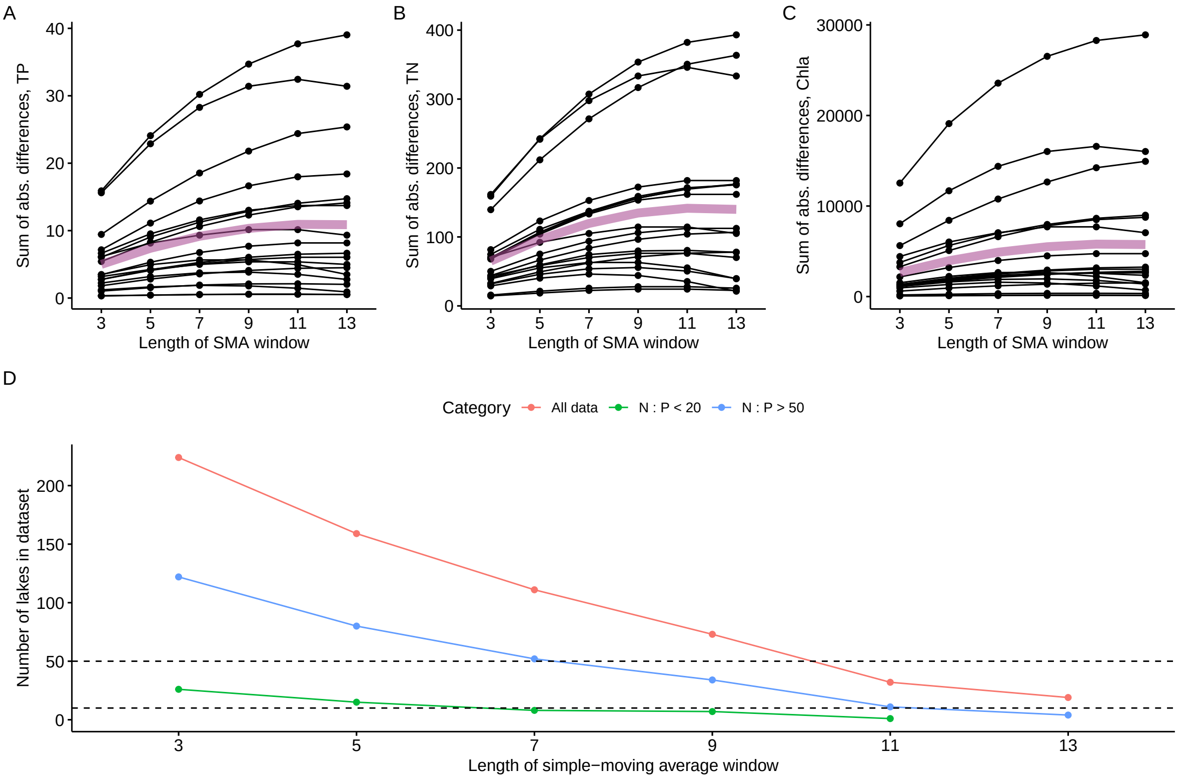

SFig. 27: Sums of absolute differences (SAD) TP (A), TN (B) and Chla (C), and number of lakes in the dataset dependent on length of SMA for all data, observations with TN : TP < 20, and observations with TN : TP > 50 (D). The horizontal dotted lines in panel D indicate 10 and 50 lakes in the dataset for visual reference.

2.5 Selecting the best regression models

2.5.1 Choosing the type of regression model

We chose generalised linear models with a Gamma link function instead of simple linear models for the 5-year SMAs because Chl-a concentrations best followed a Gamma distribution. In contrast, the residuals of the SMAs best followed a Normal distribution. We assessed this using the fitdistrplus package in R,[6] where we tested how well normal, log-normal and gamma distributions fit the data. We kept the descriptor variables as they were, so we did not apply any data transformations (log or otherwise). In both cases we used the GLM function from the GLM package in Julia, with a Gamma link function for the 5-year SMAs and a Normal link function for the SMA residuals. A Normal link GLM is equivalent to a linear model. However, we used the GLM function in both cases for maximum comparability.

2.5.2 Choice of model terms

To find the models that parsimoniously explained Chl-a concentrations, we used Akaike's information criterion (AIC)[7]. Here, we compared one-way models with TN or TP concentrations as explanatory variables for Chl-a concentrations with either additive models containing both TN and TP, or with models containing both TN and TP and an interaction term between TN and TP.

Due to the nature of the data, we calculated thousands of AIC values over the range of molar TN:TP ratios. To compare AIC values, we were not interested in their absolute values, but in the reduction of AIC by the models. To assess this, we calculated the delta AIC (ΔAIC) between models, i.e. the change in AIC by adding or removing model terms. To test the improvement in AIC for the additive models, we calculated two ΔAIC for each sample. To compare the TN+TP additive models with the TP only models, we calculated $\Delta AIC_{TP\ vs\ TN+TP}$ as follows:

$$\Delta AIC_{TP\ vs\ TN+TP} = AIC_{TP\ model} - AIC_{TN+TP\ model} \tag{4}$$

Similarly, we calculated the $\Delta AIC_{TN\ vs\ TN+TP}$ as:

$$\Delta AIC_{TN\ vs\ TN+TP} = AIC_{TN\ model} - AIC_{TN+TP\ model} \tag{5}$$

To compare the additive models (TN + TP) and models with interaction (TN * TP), we also calculated the $\Delta AIC_{TN+TP\ vs\ TN*TP}$ for each random sample:

$$\Delta AIC_{TN+TP\ vs\ TN*TP} = AIC_{TN+TP\ model} - AIC_{TN*TP\ model} \tag{6}$$

Here, a negative ΔAIC indicates a reduction in the AIC, i.e. less information is lost and the model explains the data better while being parsimonious. A ΔAIC at or above zero indicates no improvement in the model.

If we plot the $\Delta AIC_{TP\ vs\ TN+TP}$ calculated by Equation 4 against the mean molar TN:TP of the random samples for the 5-year SMAs used in the study, we find considerable variation in the response. In particular, for TN:TP < 40, we find that almost all model solutions have a negative $\Delta AIC_{TP\ vs\ TN+TP}$ (SFig. 28 A). For the $\Delta AIC_{TN\ vs\ TN+TP}$ calculated by Equation 5, we find a clear negative deviation from zero, around TN : TP = 50 (SFig. 28 B).

The distribution of ΔAIC along the TN : TP axis further supports the idea in the main text that TN and TP affected Chla differently along the TN : TP axis, and that their effects on Chla complement each other in the additive model with TN + TP.

The use of an interaction term did not improve the model quality, as indicated by the lack of deviation of $\Delta AIC_{TN+TP\ vs\ TN*TP}$ from zero (calculated by Equation 6, SFig. 28 C).

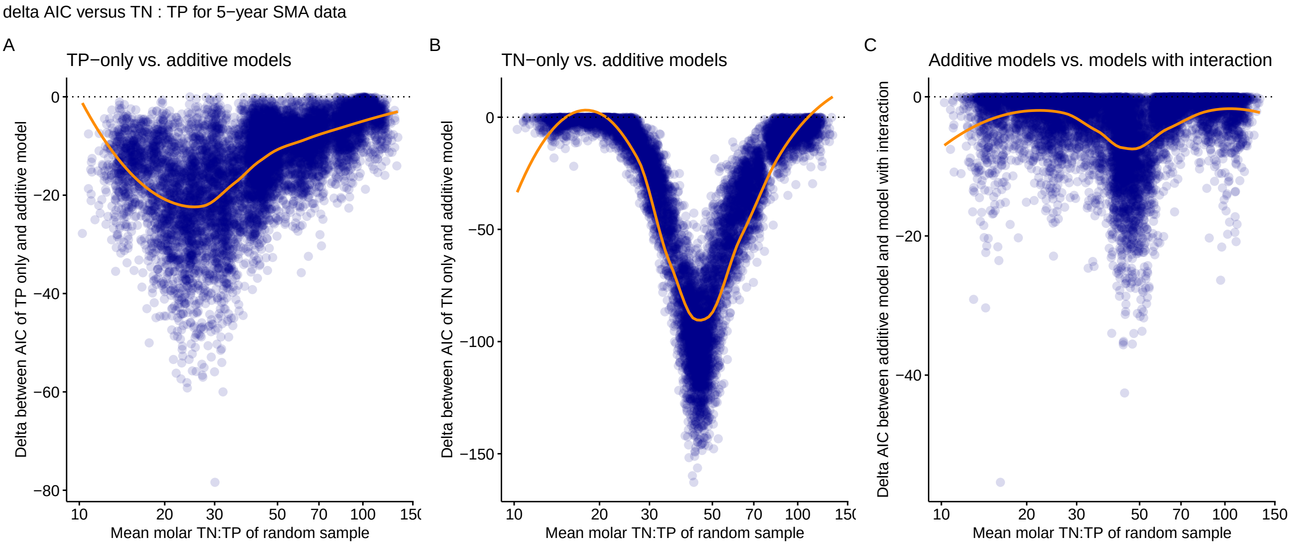

SFig. 28: Results on Delta AIC for TP-only models versus additive models (panel A) or TN-only models versus additive models (panel B), and for the Delta AIC between additive models and models with interaction term between TN and TP (panel C). The models were constructed for the 5-year simple moving averages (SMAs), therefore for lakes with 5 or more years of consecutive data. Positive values indicate an increase of the AIC (hence reduced model quality), negative values indicate a decrease of the AIC (hence increased model quality). See also Section 2.1 for details on the statistical approach. The darker the points, the more overlapping solutions were found for the AIC by the bootstrap procedure. The orange line is the average response, based on a LOESS function.

Supplementary references

1. Filazzola, A. *et al.* A database of chlorophyll and water chemistry in freshwater lakes. *Scientific Data* **7**, 310 (2020).
2. Kirillin, G. & Shatwell, T. Generalized scaling of seasonal thermal stratification in lakes. *Earth-Science Reviews* **161**, 179–190 (2016).
3. Ren, S. *et al.* Nonparametric bootstrapping for hierarchical data. *Journal of Applied Statistics* **37**, 1487–1498 (2010).
4. Svetunkov, I. & Petropoulos, F. Old dog, new tricks: A modelling view of simple moving averages. *International Journal of Production Research* **56**, 6034–6047 (2018).
5. Carpenter, S. R. & Leavitt, P. R. Temporal Variation in a Paleolimnological Record Arising from a Trophic Cascade. *Ecology* **72**, 277–285 (1991).
6. Delignette-Muller, M. L. & Dutang, C. Fitdistrplus: An R Package for Fitting Distributions. *Journal of Statistical Software* **64**, 1–34 (2015).
7. McElreath, R. *Statistical Rethinking: A Bayesian Course with Examples in R and Stan.* (CRC Press, 2018).
